# Supplementary material for: Low-Temperature-Induced Changes in Rice Panicle Architectures and Their Robustness in Extremely Cold-Tolerant Cultivars
Source: Plants (Basel). 2025 Sep 3;14(17):2759. doi: 10.3390/plants14172759 (PMC12430349; doi:10.3390/plants14172759)
Supplement: Supplementary file 1 [file plants-14-02759-s001.zip › Figures S1, S2.pdf]

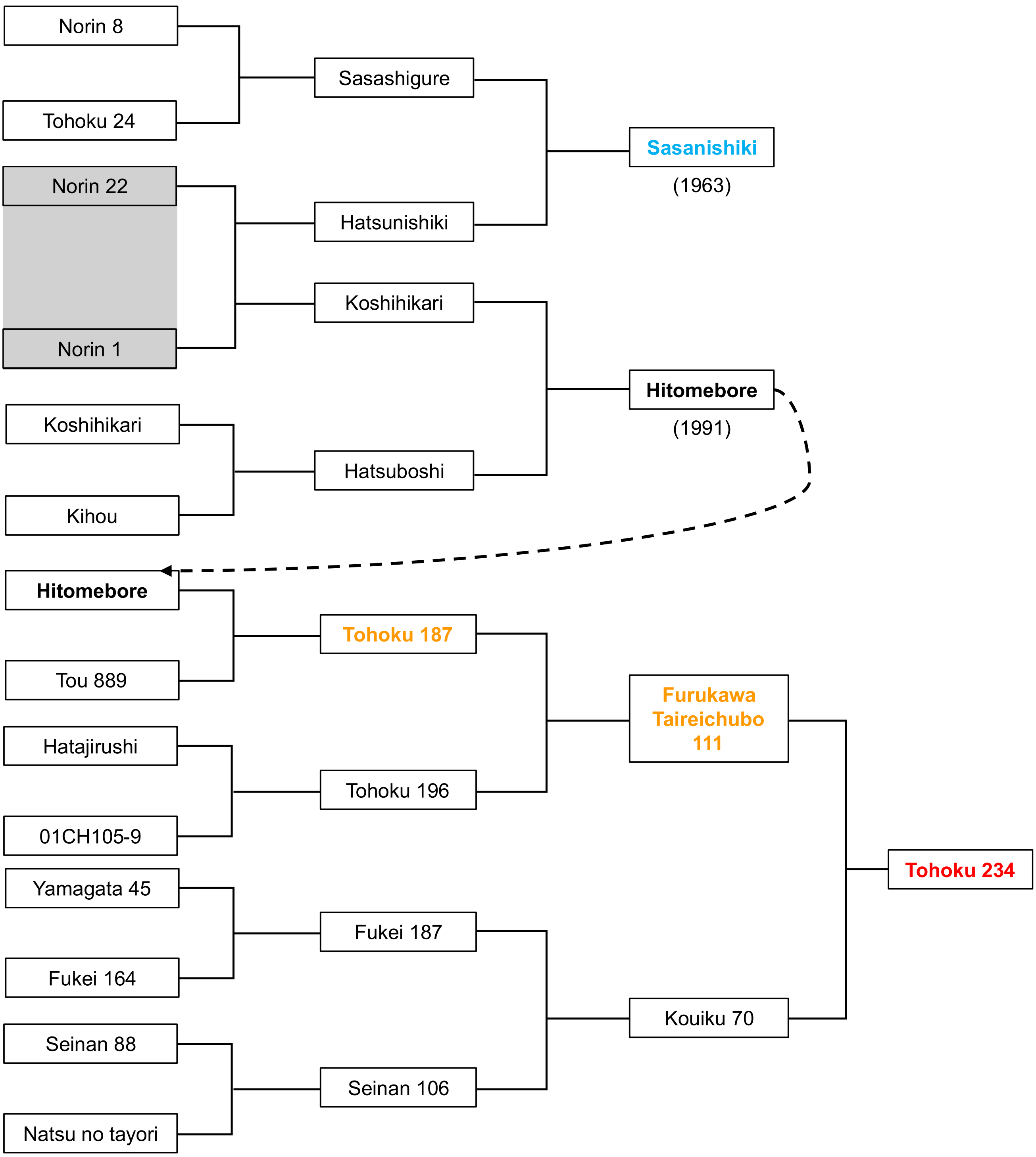

Figure S1: Breeding lineages of Sasanishiki, Hitomebore, and Tohoku 234 varieties. The figure illustrates the breeding relationships of Sasanishki, Hitomebore, and Tohoku 234 varieties used in this study. Koshihikari shares a significant genetic background with Sasanishiki and is one of the breeding parents of Hitomebore. Grey highlighted portion indicate the shared genetic background between Sasanishiki and Hitomebore. Tohoku 234 is a maternal progeny of Hitomebore with extremely strong LT and high temperature tolerance. Numbers in brackets represent variety registration year. Highlighted cultivar names are from classifications based on seed fertility under LT treatment conditions of 18.5°C in this study (see Table S1): blue: **weak**, black bold: **moderate**, orange: **strong**, red: **extremely strong**. Breeding information was retrieved from NARO website (https://www.naro.go.jp/) and translated from Japanese to English. NARO: National Agriculture and Food Research Organization.

(a)

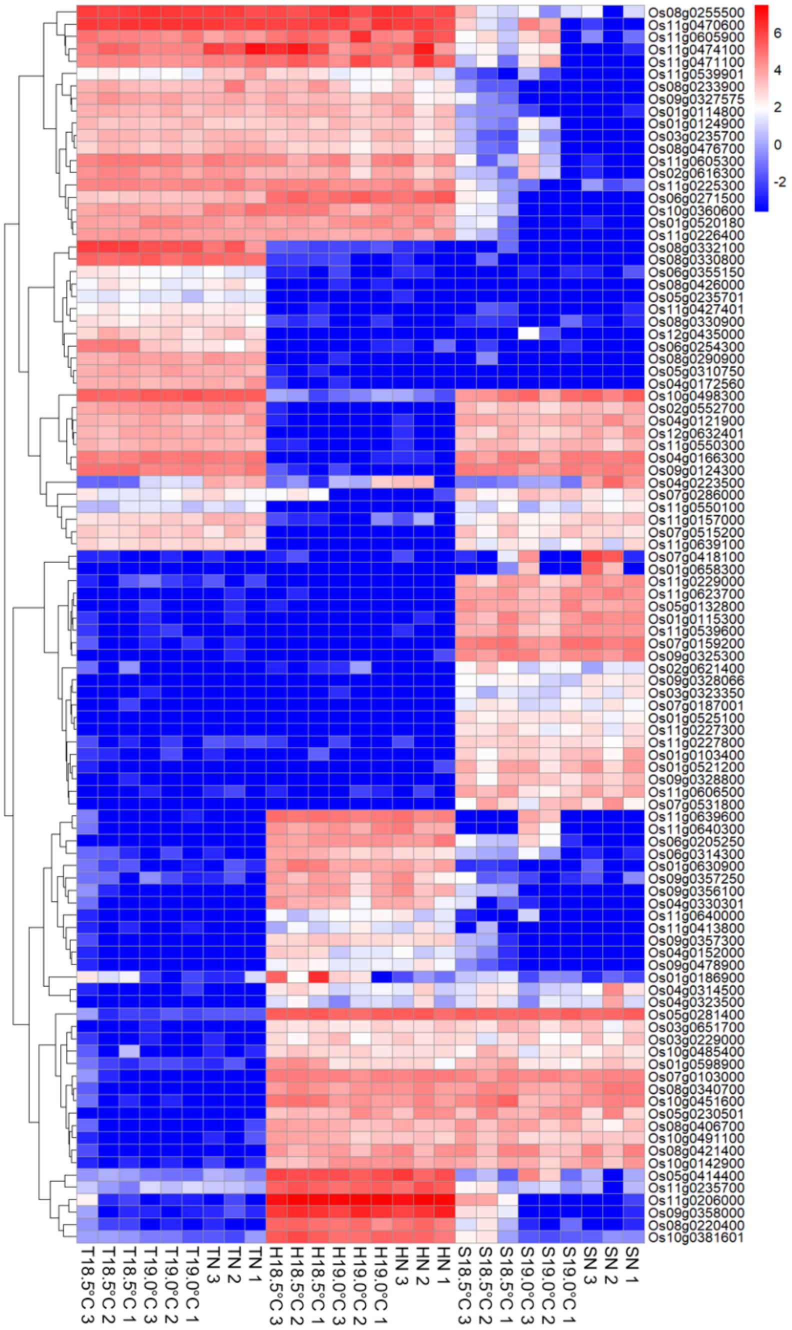
Os08g0255500
Os11g0470600
Os11g0605900
Os11g0474100
Os11g0471100
Os11g0539901
Os08g0233900
Os09g0327575
Os01g0114800
Os01g0124900
Os03g0235700
Os08g0476700
Os11g0605300
Os02g0616300
Os11g0225300
Os06g0271500
Os10g0360600
Os01g0520180
Os11g0226400
Os08g0332100
Os08g0330800
Os06g0355150
Os08g0426000
Os05g0235701
Os11g0427401
Os08g0330900
Os12g0435000
Os06g0254300
Os08g0290900
Os05g0310750
Os04g0172560
Os10g0498300
Os02g0552700
Os04g0121900
Os12g0632401
Os11g0550300
Os04g0166300
Os09g0124300
Os04g0223500
Os07g0286000
Os11g0550100
Os11g0157000
Os07g0515200
Os11g0639100
Os07g0418100
Os01g0658300
Os11g0229000
Os11g0623700
Os05g0132800
Os01g0115300
Os11g0539600
Os07g0159200
Os09g0325300
Os02g0621400
Os09g0328066
Os03g0323350
Os07g0187001
Os01g0525100
Os11g0227300
Os11g0227800
Os01g0103400
Os01g0521200
Os09g0328800
Os11g0606500
Os07g0531800
Os11g0639600
Os11g0640300
Os06g0205250
Os06g0314300
Os01g0630900
Os09g0357250
Os09g0356100
Os04g0330301
Os11g0640000
Os11g0413800
Os09g0357300
Os04g0152000
Os09g0478900
Os01g0186900
Os04g0314500
Os04g0323500
Os05g0281400
Os03g0651700
Os03g0229000
Os10g0485400
Os01g0598900
Os07g0103000
Os08g0340700
Os10g0451600
Os05g0230501
Os08g0406700
Os10g0491100
Os08g0421400
Os10g0142900
Os05g0414400
Os11g0235700
Os11g0206000
Os09g0358000
Os08g0220400
Os10g0381601
6
4
2
0
-2
T18.5°C 3
T18.5°C 2
T18.5°C 1
T19.0°C 3
T19.0°C 2
T19.0°C 1
TN 3
TN 2
TN 1
H18.5°C 3
H18.5°C 2
H18.5°C 1
H19.0°C 3
H19.0°C 2
H19.0°C 1
HN 3
HN 2
HN 1
S18.5°C 3
S18.5°C 2
S18.5°C 1
S19.0°C 3
S19.0°C 2
S19.0°C 1
SN 3
SN 2
SN 1

(b)

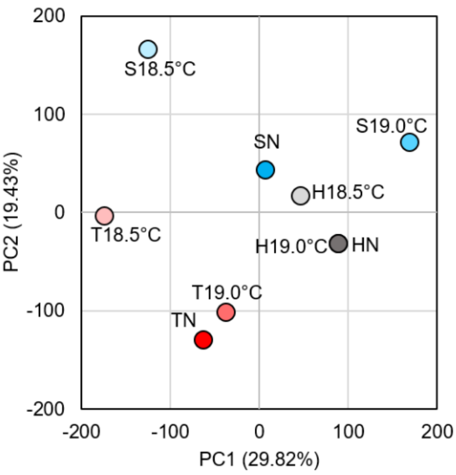
200
100
0
-100
-200
-200
-100
0
100
200
PC2 (19.43%)
PC1 (29.82%)
S18.5°C
S19.0°C
SN
H18.5°C
T18.5°C
H19.0°C
HN
T19.0°C
TN

Figure S2. Quality assessment of gene expression datasets from young panicles of Tohoku 234, Hitomebore, and Sasanishiki under different temperature conditions. (a) Heatmap of the top 100 most variable genes (ordered according to log_2CPM variance across replicates). Rows represent genes clustered by expression pattern, and columns represent biological replicates. Red, high; white, intermediate; blue, low. (**b**) Principal component analysis (PCA) of 44,823 annotated genes with an average of three replicates per sample. The first two principal components (PC1 and PC2), which accounted for the major source of variance in the expression profiles, were shown. Tohoku 234 at NT (TN), 19.0°C (T19°C), and 18.5°C (T18.5°C). Hitomebore at NT (HN), 19.0°C (H19°C), and 18.5°C (H18.5°C). Sasanishiki at NT (SN), 19.0°C (S19°C), and 18.5°C (S18.5°C).
